# Impact of meteorological variation on hospital visits of patients with tree pollen allergy

**DOI:** 10.1186/1471-2458-11-890

**Published:** 2011-11-24

**Authors:** Si-Heon Kim, Hae-Sim Park, Jae-Yeon Jang

**Affiliations:** 1 Department of Preventive Medicine and Public Health, Ajou University School of Medicine, San-5 Wonchon-dong, Youngtong-gu, Suwon, South Korea; 2 Department of Allergy and Rheumatology, Ajou University School of Medicine, San-5 Wonchon-dong, Youngtong-gu, Suwon, South Korea

## Abstract

**Background:**

Climate change could affect allergic diseases, especially due to pollen. However, there has been no epidemiologic study to demonstrate the relationship between meteorological factors, pollen, and allergic patients. We aimed to investigate the association between meteorological variations and hospital visits of patients with tree pollen allergy.

**Methods:**

The study subjects were adult patients who received skin prick tests between April and July from 1999 to 2008. We reviewed the medical records for the test results of 4,715 patients. Patients with tree pollen allergy were defined as those sensitized to more than 1 of 12 tree pollen allergens. We used monthly means of airborne tree pollen counts and meteorological factors: maximum/average/minimum temperature, relative humidity, and precipitation. We analyzed the correlations between meteorological variations, tree pollen counts, and the patient numbers. Multivariable logistic regression analyses were used to investigate the associations between meteorological factors and hospital visits of patients.

**Results:**

The minimum temperature in March was significantly and positively correlated with tree pollen counts in March/April and patient numbers from April through July. Pollen counts in March/April were also correlated with patient numbers from April through July. After adjusting for confounders, including air pollutants, there was a positive association between the minimum temperature in March and hospital visits of patients with tree pollen allergy from April to July(odds ratio, 1.14; 95% CI 1.03 to 1.25).

**Conclusions:**

Higher temperatures could increase tree pollen counts, affecting the symptoms of patients with tree pollen allergy, thereby increasing the number of patients visiting hospitals.

## Background

The global surface temperature has increased by 0.74°C over the past 100 years [1], and is projected to increase more rapidly during the next 10 decades [2], implying that climate change is likely to be a big environmental threat to health in the 21st century [3]. Several reviews [4-7] have reported that allergic diseases, especially those due to pollen, may be closely related to climate change, based on the premise that pollen amounts, allergenicity, and season are affected by climate change [8]. Some studies in different regions including Europe, America, and Asia have shown that higher temperatures are related to higher pollen counts [9-11], stronger allergenicity [12,13], as well as an earlier start and longer duration of the pollen season [9,14,15].

The prevalence of allergic diseases has rapidly increased worldwide. A previous study reported that asthma prevalence is rising in many developed and developing countries [16]. It has been suggested that the increasing prevalence of allergic diseases may be due to markedly altered environmental factors such as climate change [17]. However, there has been no appropriate epidemiologic study proving the relationship between meteorological factors, pollen, and patients allergic to pollen. A study designed to verify the impact of climate change on allergic diseases has to consider the temporal relationship that pollen counts are influenced by patterns of preseason weather, and allergic symptoms due to pollen are known to occur or worsen during or after pollen season. It is also necessary to assess association between meteorological variations and pollen allergy after adjusting other aeroallergen such as air pollution.

In the present study we investigated the possible correlations between meteorological factors, tree pollen, and allergic patients based on temporal relationship. Furthermore, we examined the extent to which variations in meteorological factors influence hospital visits of patients with tree pollen allergy while controlling for air pollution.

## Methods

### Study subjects

This study sampled patients who had allergic symptoms (asthma, rhinitis, and dermatitis) and were referred from local clinics to Ajou University Hospital between 1999 and 2008. Among them, we selected patients performed skin prick tests to identify causative allergens and reviewed their medical records for the tests. We used patients aged 18 years or older tested from April to July as study subjects. Pre-analysis allowed us to verify that hospital visits of patients sensitized to tree pollen were most prevalent during the selected study period. In Korea, total yearly counts of tree pollen are greater than those of grass or weed pollen [18]. We excluded patients without a response to a positive control or with a response to a negative control. Patients in 2001 were also excluded because many skin prick test results were missing in that year.

The study hospital is a general hospital in Suwon, a large city with a population of 1.09 million in Gyeonggi Province located near Seoul, Korea. The hospital is visited by approximately 60,000 patients per month from the city and surrounding areas. Ethical approval for the study protocol was obtained from Ajou Institutional Review Board.

### Skin prick tests

The allergens tested throughout the study period were commercial extracts from 2 manufacturers (Bencard, London, UK; Allergopharma, Reinbek, Germany). Tree pollen allergens included tree mix, alder, pine, oak, ash, beech, birch, acacia, willow, poplar, elm, and hazel. Allergens for house dust mite were used for comparison and included *Dermatophagoides pteronyssinus *and *D. farina*. Positive (histamine, 1 mg/mL) and negative (saline) controls were also applied to determine sensitization.

The skin prick test was performed by trained nurses on patients not taking interfering medicines for at least 3 days before the test. Each allergen and control solution was dropped on the patient's back. The skin was then pricked through the drops using the tip of a lancet. After 15 minutes, the lengths of the longest lines were measured together with the perpendicular bisector of the line of the wheal.

The average length of 2 lines was calculated and used as the wheal size. If the wheal size for an allergen was equal to or larger than that for the positive control the patient was considered sensitized. Patients with tree pollen allergy were defined as anyone sensitized to more than 1 of the 12 tree pollen allergens. For house dust mite, allergic patients were anyone sensitized to house dust mite allergens and not-sensitized to tree pollen allergens.

### Meteorological factors

Meteorological factors included the average, maximum, and minimum temperatures, relative humidity, and precipitation. The data were recorded daily at the meteorological observatory of Suwon and reported by the Korea Meteorological Administration. Monthly mean values were used in the analyses.

### Pollen

The present study used data recorded at the nearest pollen station, which is about 30 km distant from the study region. The pollen stations are managed by the National Institute of Meteorological Research and the Society for Pollen Research. Pollen grains were collected daily using Burkard 7 day recording volumetric spore traps (Burkard Manufacturing Co. Ltd., Rickmansworth, Hertfordshire, UK) and then counted under a microscope by reading slides prepared from the pollen-trapping tape. The pollen grains were classified as tree, grass, or weed; the tree group included pollen grains from oak, willow, hazel, poplar, elm, birch, pine, alder, juniper, plane tree, and maple. Pollen counts of each group were reported as total grains per cubic meter of air (grains/m^3^). Mean monthly counts of tree pollen were used. Pollen data in 2006 was processed into missing value since there were several days without records in that year.

### Air pollutants

This study included air pollutants as confounders since they are capable of independently causing some allergic diseases and can also modify the effect of pollen on diseases through interactions [19]. The concentrations of ozone (O_3_), particulate matter (PM_10_) and sulfur dioxide (SO_2_) in the study region were used. Daily concentrations of air pollutants were recorded at 6 air quality monitoring stations in the region and reported by Gyeonggi Air Pollution Information Center. The stations, located in areas without barriers around them such as buildings and trees, were representative of the level of air pollution in the area. The height of the samplers ranged from 1.5 to 10 m above ground level. The concentrations of O_3_, PM_10_, and SO_2 _were measured by ultraviolet photometric, β-ray absorption, and pulse ultraviolet fluorescence methods, respectively. We used the monthly average of values measured at 6 stations for each air pollutant in the analyses.

### Statistical analysis

**Figure 1:**
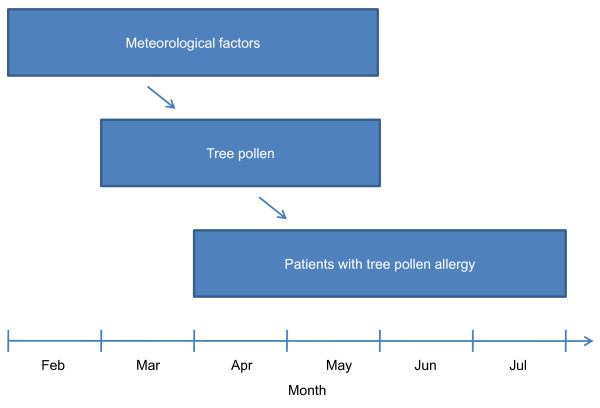
**Conceptual framework of study design**. The present study attempted to identify relationships between meteorological factors, tree pollen, and patients with tree pollen allergy based on their temporal relationship.

The present study considered lag times between meteorological factors, tree pollen, and patients with tree pollen allergy to satisfy appropriate temporal relationships (Figure 1). We used tree pollen counts from March to May since previous study reported that amount of tree pollen is very high during the period in study region [18]. We focused on patients who visited the hospital between April and July since they might be directly influenced by tree pollen. Lag times were considered from the peak of pollen exposure to the skin prick test with regards to the following: (1) the time of the initial exposure to pollen; (2) when the patient with allergic symptoms actually visited the clinic; (3) when the patient was transferred from the clinic to a general hospital by the health care delivery system in Korea; and (4) when the skin prick test was performed following a physician's order. Several studies [20-22] have shown that pollen counts are affected by meteorological factors of the preceding period, but these studies have not been consistent in the duration between the evaluated periods. In the present study we considered that pollen counts would be affected by meteorological factors in the previous or the same month. Therefore, we used meteorological factors from February to May.

Differences in age and sex in patients with and without tree pollen allergy were analyzed using the independent *t*-test and chi-square test. Pearson's correlation analysis was used to investigate correlations between meteorological factors, tree pollen counts, and the number of patients with tree pollen allergy. After selecting meteorological factors correlated with tree pollen and patients, we calculated odds ratios (ORs) of sensitization to tree pollen according to variations in meteorological factors using logistic regression analysis. Dependent variable was whether study subjects were sensitized to tree pollen or not. We also calculated ORs in the same way for house dust mite. Air pollutants, age, and sex were adjusted for in the regression analysis. Statistical analyses were performed using SPSS version 17.0 (SPSS Inc., Chicago, IL, USA) software, and a *p*-value of < 0.05 was considered significant.

## Results

**Figure 2:**
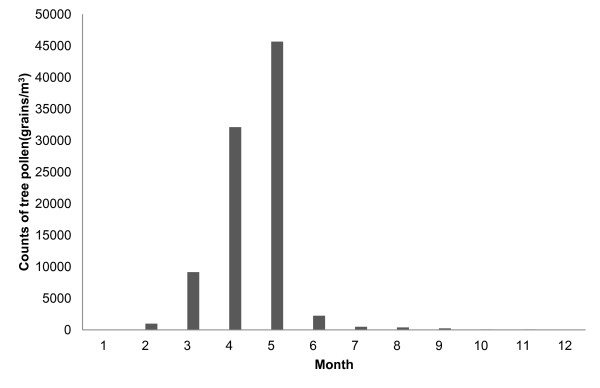
**Monthly distribution of tree pollen (1999-2008 except 2006)**. Tree pollen is most prevalent between March and May.

The final analysis included 4,715 of the 19,195 screened patients who received skin prick tests over a period of 9 years (Table 1). The mean age of the study subjects was 38.4 (±13.4 SD) years. There were 2,206 (46.8%) men and 2,509 women (53.2%). Of the test subjects, 965 patients (20.5%) had tree pollen allergy. The patients with tree pollen allergy were younger than those without it (35.4 ± 11.7 vs. 39.1 ± 13.7 years; *p *< 0.001), and the proportion of men was higher in the former group than in the latter (53.9% vs. 45.0%; *p *< 0.001). Measures of the monthly meteorological factors from 1999 to 2008 are summarized in Table 2. Tree pollen showed seasonality in that it was most prevalent from March to May, which is spring in Korea (Figure 2). About 95% of the yearly total counts of tree pollen were recorded in spring.

**Table 1:** General characteristics of the study subjects


	**Total**	**Patients with tree pollen allergy**	**Patients without tree pollen allergy**

N	4,715	965	3,750
Age (years)			
Mean	38.4	35.4	39.1
SD	13.4	11.7	13.7
Sex, men			
n	2,206	520	1,686
%	46.8	53.9	45.0

**Table 2:** Meteorological factors between February and May (1999-2008)


	**Average temperature (°C)**		**Maximum temperature (°C)**		**Minimum temperature (°C)**		**Relative humidity (%)**		**Precipitation (mm)**	

	**Mean**	**SD**	**Mean**	**SD**	**Mean**	**SD**	**Mean**	**SD**	**Mean**	**SD**

February	0.1	1.7	5.2	2.1	-4.6	1.7	58.6	6.5	0.6	0.5
March	5.6	0.7	11.1	0.9	0.5	0.7	58.9	7.0	1.2	1.3
April	12.2	0.7	18.1	1.1	6.8	0.5	59.0	4.9	2.6	1.8
May	17.4	0.7	23.1	0.7	12.4	0.6	64.8	4.8	3.2	1.0

Table 3 shows the results of correlations between meteorological factors and tree pollen counts. There were positive correlations between the minimum temperature in March and pollen counts in March and April (*p *= 0.02 and *p *= 0.005, respectively), and between relative humidity and pollen counts in March (*p *= 0.007). Pollen counts in May showed significant positive correlations with precipitation in February (*p *= 0.01) and average temperature in May (*p *= 0.04). However, no meteorological factors in April were significantly correlated with pollen counts.

**Table 3:** Correlations between mean monthly meteorological factors and tree pollen counts


**Month**	**Meteorological factors**	**Counts of tree pollen (grains/m^3^)**		
		
		**March**	**April**	**May**

February	Average temperature (°C)	0.04	-0.16	0.22
	Maximum temperature (°C)	0.08	-0.08	0.15
	Minimum temperature (°C)	-0.02	-0.27	0.25
	Relative humidity (%)	0.24	-0.02	0.16
	Precipitation (mm)	0.06	-0.04	0.82*
March	Average temperature (°C)	0.50	0.62	-0.10
	Maximum temperature (°C)	0.17	0.43	-0.37
	Minimum temperature (°C)	0.78*	0.86*	0.16
	Relative humidity (%)	0.85*	0.65	0.13
	Precipitation (mm)	0.50	0.24	-0.11
April	Average temperature (°C)	-	0.48	0.11
	Maximum temperature (°C)	-	0.45	-0.11
	Minimum temperature (°C)	-	0.34	0.44
	Relative humidity (%)	-	0.24	0.07
	Precipitation (mm)	-	0.01	0.68
May	Average temperature (°C)	-	-	0.72*
	Maximum temperature (°C)	-	-	0.64
	Minimum temperature (°C)	-	-	0.61
	Relative humidity (%)	-	-	-0.47
	Precipitation (mm)	-	-	-0.03

Data are Pearson's correlation coefficients. *P < 0.05

The results of correlations between tree pollen counts and patients with tree pollen allergy are shown in Table 4. The pollen counts in March and April were positively correlated with the number of patients in April/May/June and April/May/July, respectively. In addition, pollen counts in March and April were positively correlated with the total number of patients who visited hospital from April to July (*p *= 0.008 and *p *= 0.003, respectively). However, pollen counts in May did not significantly correlate with either the monthly or total number of patients.

**Table 4:** Correlations between tree pollen counts and the numbers of patients with tree pollen allergy


**Counts of tree pollen (grains/m^3^)**	**No. of patients**				
	
	**April**	**May**	**June**	**July**	**Total**

March	0.86*	0.86*	0.74*	0.60	0.84*
April	0.92*	0.88*	0.59	0.85*	0.89*
May	0.38	0.42	0.44	0.61	0.50

Data are Pearson's correlation coefficients. *P < 0.05

Table 5 shows the results of correlations between meteorological factors and patients with tree pollen allergy. Minimum temperature in March was significantly correlated with monthly numbers of patients in April/May/June and total numbers of patients from April to July. None of the meteorological factors in February, April, or May showed significant correlations with patient numbers.

**Table 5:** Correlations between mean monthly meteorological factors and the number of patients with tree pollen allergy


**Month**	**Meteorological factors**	**No. of patients**				
		
		**April**	**May**	**June**	**July**	**Total^a^**

February	Average temperature (°C)	-0.21	0.03	0.56	-0.08	0.06
	Maximum temperature (°C)	-0.14	0.06	0.59	-0.04	0.11
	Minimum temperature (°C)	-0.29	-0.02	0.49	-0.14	-0.01
	Relative humidity (%)	-0.12	-0.007	0.21	-0.26	-0.05
	Precipitation (mm)	0.08	0.24	0.44	0.38	0.30
March	Average temperature (°C)	0.39	0.38	0.37	0.24	0.38
	Maximum temperature (°C)	0.15	0.11	0.05	0.07	0.11
	Minimum temperature (°C)	0.71*	0.76*	0.68*	0.56	0.75*
	Relative humidity (%)	0.63	0.57	0.54	0.25	0.56
	Precipitation (mm)	0.27	0.35	0.60	-0.03	0.33
April	Average temperature (°C)	0.33	0.18	-0.14	0.35	0.21
	Maximum temperature (°C)	0.30	0.14	-0.16	0.24	0.15
	Minimum temperature (°C)	0.23	0.24	-0.02	0.37	0.22
	Relative humidity (%)	0.16	0.29	0.25	-0.10	0.16
	Precipitation (mm)	-0.03	-0.11	-0.12	0.28	-0.005
May	Average temperature (°C)	-	0.54	0.47	0.36	0.5
	Maximum temperature (°C)	-	0.53	0.38	0.34	0.45
	Minimum temperature (°C)	-	0.36	0.45	0.22	0.38
	Relative humidity (%)	-	-0.1	0.03	-0.44	-0.18
	Precipitation (mm)	-	0.26	0.55	-0.01	0.3

Data are Pearson's correlation coefficients. *P < 0.05

^a^Total numbers of patients are the sum of patients with tree pollen allergy from April through July. For meteorological factors in May, total numbers of patients from May to July were used

**Figure 3:**
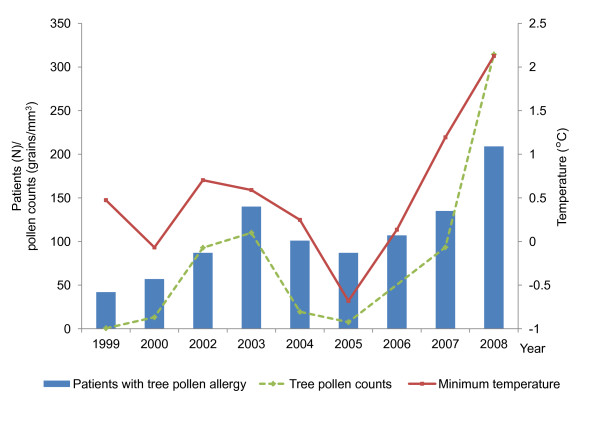
**Annual trends of minimum temperature, tree pollen counts, and patients with tree pollen allergy**. Value for each variable is following; monthly mean value of minimum temperature in March for minimum temperature (solid line), monthly mean value of tree pollen counts in March and April for tree pollen counts (dashed line), and total numbers of tree pollen-sensitized patients visited in hospital from April through July for patients with tree pollen allergy (bar graph).

Considering the correlation results comprehensively, the minimum temperature in March correlated with tree pollen counts in March and April, as did the latter with the number of patients with tree pollen allergy. The minimum temperature in March also correlated with the number of patients. Annual trends of minimum temperature, tree pollen counts, and patients with tree pollen allergy showed similar patterns (Figure 3). Consequently, there were positive and temporal relationships between temperature, tree pollen counts, and the number of patients with tree pollen allergy.

On the basis of our previous results, we examined the effects of minimum temperature in March on hospital visits of patients with tree pollen allergy (Table 6). In unadjusted analysis, hospital visits of patients significantly increased with a rise in temperature (OR, 1.12; 95% CI, 1.03 to 1.21). In age- and sex-adjusted analysis (Model 1), there was still a positive association between temperature and hospital visits (OR, 1.11; 95% CI, 1.02 to 1.21). Even after additionally adjusting for air pollutants (Model 2), the association remained significant (OR, 1.14; 95% CI, 1.03 to 1.25). These findings may indicate that a rise of 1°C in the minimum temperature in March can increase the proportion of patients with tree pollen allergy by 14% of the total patients tested between April and July. In the analysis for house dust mite as control, 4,131 patients were included after excluding 584 patients sensitized to tree pollen and house dust mite. In contrast to the findings for tree pollen allergy, hospital visits of patients with house dust mite allergy were not significantly associated with the minimum temperature in March.

**Table 6:** Associations between the minimum temperature in March and hospital visits of patients with tree pollen and house dust mite allergy


	**Patients with tree pollen allergy**		**Patients with house dust mite allergy**	
	
	**OR**	**95% CI**	**OR**	**95% CI**

Unadjusted	1.12	1.03 to 1.21	1.03	0.95 to 1.12
Model 1^a^	1.11	1.02 to 1.21	1.01	0.93 to 1.10
Model 2^b^	1.14	1.03 to 1.25	0.99	0.90 to 1.09

^a^Adjusted for age and sex. ^b^Adjusted for age, sex, and air pollutants (O_3_, PM_10_, and SO_2_). Concentrations of each air pollutant were the mean values of those from March to April

## Discussion

The present study identified strong, positive, and significant correlations with temporal relationships between meteorological factors, tree pollen, and patients with tree pollen allergy. It was confirmed that the number of patients with tree pollen allergy who visited the hospital from April through July was correlated with tree pollen counts in March and April, and the minimum temperature in March was the only meteorological factor found to correlate with tree pollen and allergic patients. The other meteorological factors also correlated with tree pollen counts, but did not show any correlation with the number of allergic patients.

The patterns of hospital visits of patients with tree pollen allergy may be more affected by factors other than changes in pollen counts following meteorological variations. There is a possibility that these patterns resulted from the effects of other environmental or socio-demographic factors, e.g., air pollution, change in the recognition of allergic diseases, public image of the study hospital, the population number in the study region, etc. However, a higher minimum temperature in March was associated with increased hospital visits of patients sensitized to tree pollen even after controlling for air pollutants, age, and sex. Moreover, patients allergic to house dust mite, another major allergen in Korea, did not show an association with the minimum temperature in March. Therefore, these findings suggest that there could be a possible causal relationship between increasing temperature and higher tree pollen counts, thereby leading to more patients sensitized to tree pollen and consequently more visits to the hospital.

Some patients with tree pollen allergy in this study could be multi-sensitized to various allergens. It is appropriate to use patients sensitized to tree pollen alone for drawing more definite results in the analysis. However, it is impossible to perform skin prick test for all of known allergen to rule out multi-sensitized patients. Although patients allergic to tree pollen are multi-sensitized, they could be exposed to tree pollen judging from the timing of hospital visit, and lead to aggravate allergic symptoms.

Previous studies have reported relationships between several meteorological factors and pollen: temperature, relative humidity, and pollen counts [22,23], temperature, precipitation and pollen counts [18], temperature and the start of the pollen season [21,24], and temperature and allergenicity [12]. Temperature is a common meteorological factor influencing pollen dynamics in these studies, consistent with our results showing that temperature is strongly correlated with pollen counts. Some studies [25-27] reported that advance of flowering was correlated with increase in minimum temperature, which suggests that minimum temperature could influence pollen production.

Only a few studies have investigated the relationship between temperature and allergic diseases. Hales et al. [28] showed that there is a positive association between mean temperature and the prevalence of asthma symptoms, and similar results were reported by Zanolin et al. [29]. In contrast, another study reported that the prevalence of atopic eczema is positively associated with precipitation and humidity, and is negatively associated with temperature and the number of sunny hours [30]. However, these studies had limitations in that the causative allergens, such as pollen, were not included in the analyses; therefore, they may not reflect a chain of processes from climate change to pollen allergic diseases.

Arino et al. [31] reported progressive increases in the values of some meteorological factors including temperature, the seasonal duration of pollen, and the percentage of patients sensitized to pollens; they also observed correlations between the variables. However, they did not control for confounders and investigate their possible associations. In addition, because the study used year-by-year data values, it did not considered how pollen counts or seasons would be affected by meteorological factors during blossoming or the period immediately prior to blossoming.

The majority of the previous studies have been ecological in nature and evaluated two variables such as meteorological factors and pollen or allergic patients. These methods are not sufficient to investigate the extent to which meteorological variations affect allergic diseases. The present study is the first to demonstrate the impact of temperature variation on pollen allergies based on the temporal relationships between meteorological factors, pollen counts, and patients with pollen allergy. In many of the previous studies, study subjects may have been sensitized to different species of allergens since the studies did not identify the exact allergens using objective tests. In the present study, we reviewed the results of skin prick tests to confirm the causative etiology of the allergies; thus, we were able to use well-defined patients as subjects. We also statistically controlled for the confounding effects of air pollution that is capable of causing allergic diseases and/or affecting pollen. Those points are important in the respect that our approach was based on the possible mechanism of an increased burden of allergic diseases as a result of climate change.

The present study is not without limitations. First, it is difficult to generalize our results to the general population as a whole because most of the patients in the general hospital have severe symptoms. However, it was inevitable to use hospital data to acquire a large pool of allergic patients that had undergone skin prick tests. Second, there may be uncertainties as a result of some missing patient and pollen information, and of some disparity between the species of tree pollen used in the skin prick tests and the pollen data.

## Conclusions

Our findings suggest that temperature, pollen counts, and the number of patients with pollen allergies could be closely related. Furthermore, anticipated climate change could increase the burden of allergic diseases. To confirm the relationships more precisely, further studies are needed using large population-based cohorts over longer time periods, as well as further investigation of the effects of meteorological factors on allergic diseases due to other pollen types such as those from grass and weed.

## Competing interests

The authors declare that they have no competing interests.

## Authors' contributions

SHK has been involved in data acquisition, ran the statistical analysis and wrote the first draft. SHK was also involved in the interpretation of data and in the revision of the manuscript. HSP have made substantial contribution to data acquisition and to the revision. JYJ has designed the study, interpreted the statistical results and been involve in the revision. All authors read and approved the final manuscript.

## Pre-publication history

The pre-publication history for this paper can be accessed here:

http://www.biomedcentral.com/1471-2458/11/890/prepub

## References

[B1] Trenberth KE Jones PD Adler R Alexander L Alexandersson H Allan R Baldwin MP Solomon S, Qin D, Manning M, Chen Z, Marquis M, Averyt KB, Tignor M, Miller HL Observations: surface and atmospheric climate change Climate change 2007: the physical scinece basis Contribution of the Working Group I to the Fourth Assessment Report of the Intergovernmental Panel on Climate Change 2007 Cambridge: Cambridge University Press 236 336

[B2] Meehl GA Stocker TF Collins WD Friedlingstein P Gaye AT Gregory JM Kitoh A Solomon S, Qin D, Manning M, Chen Z, Marquis M, Averyt KB, Tignor M, Miller HL Global climate projections Climate change 2007: the physical scinece basis Contribution of the Working Group I to the Fourth Assessment Report of the Intergovernmental Panel on Climate Change 2007 Cambridge: Cambridge University Press 748 845

[B3] Costello A Abbas M Allen A Ball S Bell S Bellamy R Friel S Groce N Johnson A Kett M Lee M Levy C Maslin M McCoy D McGuire B Montgomery H Napier D Pagel C Patel J de Oliveira JA Redclift N Rees H Rogger D Scott J Stephenson J Twigg J Wolff J Patterson C Managing the health effects of climate change: Lancet and University College London Institute for Global Health Commission Lancet 2009 373 1693 1733 10.1016/S0140-6736(09)60935-1 19447250

[B4] Beggs PJ Bambrick HJ Is the global rise of asthma an early impact of anthropogenic climate change? Environ Health Perspect 2005 113 915 919 10.1289/ehp.7724 16079058 PMC1280328

[B5] D'Amato G Cecchi L Bonini S Nunes C Annesi-Maesano I Behrendt H Liccardi G Popov T van Cauwenberge P Allergenic pollen and pollen allergy in Europe Allergy 2007 62 976 990 10.1111/j.1398-9995.2007.01393.x 17521313

[B6] Reid CE Gamble JL Aeroallergens, allergic disease, and climate change: impacts and adaptation Ecohealth 2009 6 458 470 10.1007/s10393-009-0261-x 19908096 PMC2880235

[B7] Shea KM Truckner RT Weber RW Peden DB Climate change and allergic disease J Allergy Clin Immunol 2008 122 443 453 10.1016/j.jaci.2008.06.032 18774380

[B8] Beggs PJ Impacts of climate change on aeroallergens: past and future Clin Exp Allergy 2004 34 1507 1513 10.1111/j.1365-2222.2004.02061.x 15479264

[B9] Frei T Gassner E Climate change and its impact on birch pollen quantities and the start of the pollen season an example from Switzerland for the period 1969-2006 Int J Biometeorol 2008 52 667 674 10.1007/s00484-008-0159-2 18481116

[B10] Kishikawa R Koto E Iwanaga T So N Kamori C Shoji S Nishima S Ishikawa T Long-term study of airborne allergic pollen count, C. Japonica and cupressaceae in Japan Arerugi 2001 50 369 378 11398333

[B11] Sahney M Chaurasia S Seasonal variations of airborne pollen in Allahabad, India Ann Agric Environ Med 2008 15 287 293 19061265

[B12] Ahlholm JU Helander ML Savolainen J Genetic and environmental factors affecting the allergenicity of birch (*Betula pubescens *ssp. *czerepanovii *[Orl.] Hämet-ahti) pollen Clin Exp Allergy 1998 28 1384 1388 10.1046/j.1365-2222.1998.00404.x 9824411

[B13] Hjelmroos M Schumacher MJ Van Hage-Hamsten M Heterogeneity of pollen proteins within individual *Betula pendula *trees Int Arch Allergy Immunol 1995 108 368 376 10.1159/000237184 7580310

[B14] Emberlin J Detandt M Gehrig R Jaeger S Nolard N Rantio-Lehtimäki A Responses in the start of *Betula *(birch) pollen seasons to recent changes in spring temperatures across Europe Int J Biometeorol 2002 46 159 170 10.1007/s00484-002-0139-x 12242471

[B15] Teranishi H Kenda Y Katoh T Kasuya M Oura E Taira H Possible role of climate change in the pollen scatter of Japanese cedar *Cryptomeria japonica *in Japan Clim Res 2000 14 65 70

[B16] Eder W Ege MJ von Mutius E The asthma epidemic N Engl J Med 2006 355 2226 2235 10.1056/NEJMra054308 17124020

[B17] Mösges R The increasing prevalence of allergy: a challenge for the physician Clin Exp All Rev 2002 2 13 17 10.1046/j.1472-9725.2002.00029.x

[B18] Oh JW Development of pollen concentration prediction models J Korean Med Assoc 2009 52 579 591 10.5124/jkma.2009.52.6.579

[B19] D'Amato G Liccardi G D'Amato M Cazzola M Outdoor air pollution, climatic changes and allergic bronchial asthma Eur Respir J 2002 20 763 776 10.1183/09031936.02.00401402 12358357

[B20] Frei T The effects of climate change in Switzerland 1969-1996 on airborne pollen quantities from hazel, birch and grass Grana 1998 37 172 179 10.1080/00173139809362662

[B21] Rasmussen A The effects of climate change on the birch pollen season in Denmark Aerobiologia 2002 18 253 265 10.1023/A:1021321615254

[B22] Weryszko-Chmielewska E Puc M Piotrowska K Effect of meteorological factors on *Betula*, *Fraxinus *and *Quercus *pollen concentrations in the atmosphere of Lublin and Szczecin, Poland Ann Agric Environ Med 2006 13 243 249 17195996

[B23] Bartková-Ščevková J The influence of temperature, relative humidity and rainfall on the occurrence of pollen allergens (*Betula*, *Poaceae*, *Ambrosia artemisiifolia*) in the atmosphere of Bratislava (Slovakia) Int J Biometeorol 2003 48 1 5 10.1007/s00484-003-0166-2 12690548

[B24] Yli-Panula E Fekedulegn DB Green BJ Ranta H Analysis of airborne *betula *pollen in Finland; a 31-year perspective Int J Environ Res Public Health 2009 6 1706 1723 10.3390/ijerph6061706 19578456 PMC2705213

[B25] Abu-Asab MS Peterson PM Shetler SG Orli SS Earlier plant flowering in spring as a response to global warming in the Washington, DC, area Biodivers Conserv 2001 10 597 612 10.1023/A:1016667125469

[B26] Bower JE Has climatic warming altered spring flowering date of Sonoran Desert shrubs? Southeast Nat 2007 52 347 355 10.1894/0038-4909(2007)52[347:HCWASF]2.0.CO;2

[B27] Fleming TH Sahley CT Holland JN Nason JD Hamrick JL Sonoran desert columnar cacti and the evolution of generalized pollination system Ecol Monogr 2001 71 511 530 10.1890/0012-9615(2001)071[0511:SDCCAT]2.0.CO;2

[B28] Hales S Lewis S Slater T Crane J Pearce N Prevalence of adult asthma symptoms in relation to climate in New Zealand Environ Health Perspect 1998 106 607 610 10.1289/ehp.98106607 9722625 PMC1533139

[B29] Zanolin ME Pattaro C Corsico A Bugiani M Carrozzi L Casali L Dallari R Ferrari M Marinoni A Migliore E Olivieri M Pirina P Verlato G Villani S Marco R The role of climate on the geographic variability of asthma, allergic rhinitis and respiratory symptoms: results from the Italian study of asthma in young adults Allergy 2004 59 306 314 10.1046/j.1398-9995.2003.00391.x 14982513

[B30] Suárez-Varela MM García-Marcos Alvarez L Kogan MD González AL Gimeno AM Aguinaga Ontoso I Díaz CG Pena AA Aurrecoechea BD Monge RM Quiros AB Garrido JB Canflanca IM Varela AL Climate and prevalence of atopic eczema in 6- to 7-year-old school children in Spain. ISAAC phase III Int J Biometeorol 2008 52 833 840 10.1007/s00484-008-0177-0 18779981

[B31] Ariano R Canonica GW Passalacqua G Possible role of climate changes in variations in pollen seasons and allergic sensitizations during 27 years Ann Allergy Asthma Immunol 2010 104 215 222 10.1016/j.anai.2009.12.005 20377111

